# IGF-1 downregulates matrix metalloproteinase 8 to promote plaque stability: Evidence from myeloid cell-specific MMP8 in atherosclerosis

**DOI:** 10.1371/journal.pone.0332660

**Published:** 2025-09-24

**Authors:** Patricia Snarski, Sergiy Sukhanov, Tadashi Yoshida, Svitlana Danchuk, Andrew Gross, Fareed Sindi, Vikara Rivera-Lopez, Patrice Delafontaine, Yusuke Higashi

**Affiliations:** 1 Section of Cardiology, John W. Deming Department of Medicine, Tulane University School of Medicine, New Orleans, Louisiana, United States of America; 2 Department of Physiology, Tulane University School of Medicine, New Orleans, Louisiana, United States of America; 3 Department of Pharmacology, Tulane University School of Medicine, New Orleans, Louisiana, United States of America; 4 Neuroscience, Tulane University, New Orleans Louisiana, United States of America; 5 Department of Pathology, Tulane University School of Medicine, New Orleans, Louisiana, United States of America; 6 Loyola University, New Orleans, Louisiana, United States of America; 7 VA Medical Center, New Orleans, Louisiana, United States of America; University of Vermont College of Medicine, UNITED STATES OF AMERICA

## Abstract

**Background & aims:**

Macrophages (MF) play an important role in atherosclerosis, a chronic inflammatory disease. Matrix metalloproteinase 8 (MMP8), a collagen degrading enzyme, is expressed by inflammatory cells. Systemic MMP8 deficiency reduces plaque MF and increases collagen suggesting increased plaque stability, however contribution of MF specific MMP8 is unknown. We previously found in Apolipoprotein E^-/-^ mice, Insulin-Like Growth Factor-1 (IGF-1) overexpression in MF reduced MMP8, decreased atherosclerotic plaque MF, and upregulated features of stable atherosclerotic plaque. Thus, we hypothesized that MF specific MMP8 deficiency would reduce plaque burden and promote stability.

**Methods:**

We used human THP-1 and murine MMP8 deficient MF for in vitro investigation of IGF-1 effect. We generated mice with MF MMP8-deficiency (mM8-:M8 + mice) or MF MMP8-rescue (mM8+ :M8-) by bone marrow transplantation after irradiation; IGF-1 was administered to these and control mice.

**Results:**

We found IGF-1 reduced MMP8 and suppressed collagenase activity in cultured MF. In Apolipoprotein E^-/-^ mice, MF specific IGF-1 overexpression decreased plaque MMP8 levels and pro-inflammatory cytokines. MMP8 deficient MF had decreased levels of M1 markers and increased expression of M2 markers. We found no difference in atherosclerotic burden between groups, moreover, the ability of IGF-1 to increase collagen production depends on the ability of macrophages to express MMP8. This *in vivo* effect was only found in females.

**Conclusions:**

IGF-1 downregulated plaque MMP8 levels in control mice, however this effect was markedly blunted in mM8-:M8 + mice showing that macrophages are the main target of IGF-1 downregulation of plaque MMP8. Overall, our results suggest that macrophage MMP8 may be a potential target to treat unstable atherosclerotic plaques.

## Introduction

Cardiovascular disease (CVD) remains the leading cause of death globally with atherosclerosis being the primary cause of CVD [1]. Although plaque burden contributing to the narrowing of arteries was historically of high clinical concern, there is a large body of work identifying plaque stability as a key indication of adverse events [2]. Plaque stability, which results in a thicker fibrotic cap requires several mechanisms to prevent rupture [3]. Plaque rupture or erosion are the most common mechanisms leading to acute ischemic cardiovascular events [4–6]. Monocytes and macrophages (MF) predominantly regulate the inflammatory response that mediates atherosclerotic disease progression [7–9]. MF enter the plaque in both early and late-stage atherosclerosis, although the exact role and process may contribute differently to atheroprogression [10,11]. As plaque progresses, MF both proliferate in and are recruited to the plaque by chemokines and polarize into a number of phenotypes. Traditionally, macrophages were classified as proinflammatory M1 macrophages or reparative or resolving M2 macrophages [12,13]. More inflammatory macrophages contribute to the size and destabilization of the plaque [11]. However, recent advancements in macrophage phenotyping have highlighted several additional M1/M2-like macrophage subsets, such as Mox, M4, M(Hb) and Mhem, all that have mixed beneficial and adverse effects [14]. Despite the broader diversity of MF phenotypes [15], the M1/M2 classification is still useful for a general understanding of macrophage behavior, and these phenotypes do indeed exist in both mouse and human plaque [11]. M1 macrophages are the predominant phenotype in atherosclerotic plaque, although other populations play important roles [16].

Importantly, M1 macrophages, upon activation, express matrix metalloproteinase (MMPs) including matrix metalloproteinase 8 (MMP8) [17,18]. MMPs are zinc dependent proteases that have significant roles in development but also in metabolic disorders; their presence is often pathological. MMP8 mediates degradation of collagen (a major substrate is collagen type I) [18,19], induces the cleavage of angiotensin I to produce angiotensin II [18], and is also involved in macrophage differentiation and polarization [20]. Whether in mouse or human, the major source of collagen that makes up a stable fibrotic cap comes from smooth muscle cells that have migrated from the media to the intima [21]. This deposition of collagen is limited by the activity and expression of MMPs. MF are the main source and regulator of MMP expression and thereby collagen deposition [22]. MMPs are a large family, but only MMP1, 8, and 13 initiate breakdown of intact collagen, with MMP8 having a 3 fold higher enzymatic activity for type I collagen than the other interstitial collagenases [23–26]. Once cleaved, various MMPs, such as 2, 3, and 9 further degrade collagen fibers [27]. Hermann et al. found that endothelial cells, smooth muscle cells, and macrophages all express MMP8, whilst macrophages are the major source of MMP expression [23]. Laxton et al. showed that murine global MMP8 knock out resulted in increased collagen and reduced atherosclerotic burden, however the contribution of macrophage-specific MMP8 to these effects is unknown. MMP8 has an increased catalytic efficiency of 3–4000 fold to digest type I collagen compared to MMP1 or 2 [26,28]. This may come from several structural distinctions where MMP8 has differences in substrate binding compared to other MMPs [29]. Further, increased plaque MMP8 level from patients undergoing carotid endarterectomy are associated with an increased risk of systemic CVD, while MMP2 and MMP9 had no predictive power. Moreover, macrophages presence was elevated, and collagen was decreased in plaque in patients with high MMP8, while smooth muscle content and vessel density remained unchanged [30]. Therefore, we aimed to test whether MMP8 deficiency specifically in MF would affect atherosclerosis.

Our lab has shown pluripotent effects of Insulin-like growth factor 1 (IGF-1) in atheroprotection, including reduction of MMP8 in macrophages [31]. IGF-1 is a pro-survival growth factor that is continually and almost ubiquitously expressed throughout life [32]. IGF-1 derived from the liver exerts endocrine effects, playing a critical role in normal growth and development [33,34]. However, many IGF-1 mediated effects result from autocrine and paracrine mechanisms as most cells express both IGF-1 and its receptor, Insulin-like growth factor 1 receptor (IGF-1R) [35]. There is growing evidence that circulating IGF-1 levels are inversely related to the risk of CVD [35–37]. Using an Apolipoprotein E deficient (Apoe^-/-^) mouse model of atherosclerosis, we have previously demonstrated that IGF-1 exerts anti-atherosclerotic effects in multiple cell types and via multiple mechanisms [38–41]. Systemic administration of IGF-1 (via osmotic mini pumps), and locally produced IGF-1 demonstrate anti-atherosclerotic effects [31,41]. Deficiency of monocyte/macrophage-specific IGF1R resulted in increased atherogenesis, reduced plaque collagen content, increased inflammatory monocytes in the circulation, and increased expression of MMPs in macrophages [38]. Notably, we have recently demonstrated that MF-specific IGF-1 overexpression leads to a reduction in atherosclerosis, a reduction in necrotic core, an increase in collagen and other features of plaque stability, and a decrease of MF MMPs, including MMP8 [31]. MF-specific IGF-1 overexpression mice have increased plaque collagen; however, mechanisms of increased collagen remain unclear.

We designed this study to 1) investigate the role of MF MMP8 in early and intermediate development and stability of atherosclerotic plaque and 2) test whether IGF-1-induced effect on atherosclerotic plaque depends on MF-derived MMP8. We administered IGF-1 (or saline, control) to atherosclerotic mice with cell specific MMP8 deficiency. We found that IGF-1 administration downregulated plaque MF and MMP8 levels in control and mM8 + :M8- mice, however this effect was markedly blunted in mM8-:M8 + mice showing that MMP8 expressed in MFs is the major mediator of the IGF-1 effect on plaque development and progression. Our results establish the importance of MF MMP8 as a potential target to treat unstable atherosclerosis.

## Results

### IGF-1 downregulates MMP8 and reduces collagenase activity in cultured human MF

We have reported that MF-specific IGF-1 overexpression downregulated MMP8 levels in peritoneal MF, reduced plaque MF content and decreased atherosclerotic burden [31]. These data suggest that a possible mechanism of IGF-1 atheroprotection involves MMP8 modulation of MF behavior within the plaque.

**Fig 1:**
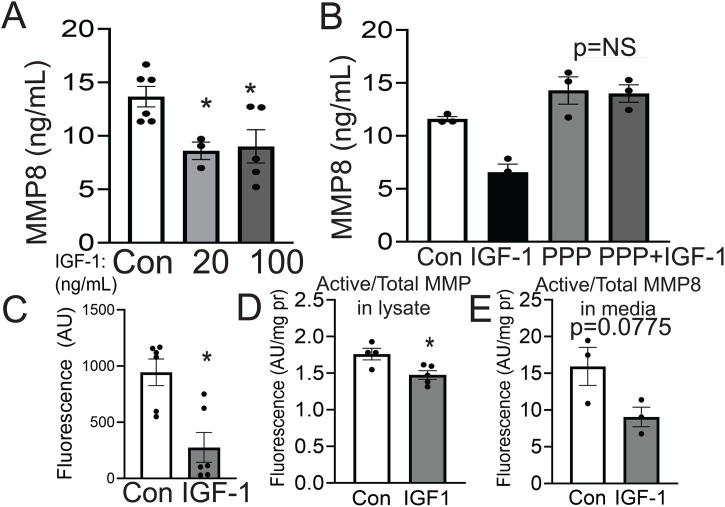
IGF-1 downregulates MMP8 and reduces collagenase activity in cultured THP-1 macrophages. **A)** THP-1 macrophages were differentiated with PMA (100nM, 48 hours), serum starved, and treated with IGF-1 (20, 100 ng/mL for 48 hours). MMP8 levels were measured in the culture supernatant by ELISA. (n = 3−6 wells in two independent experiments). **B)** THP-1 macrophages were treated with 100ng/mL IGF-1 and/or picropodophyllin (PPP, 50nM) for 48 hours and MMP8 was measured in the culture supernatant by ELISA. (n = 3 wells per group). **C)** After THP-1 differentiation, cells were treated for 24 hours with 100ng/mL IGF-1, and DQ fluorescent collagen (50 µg/mL) was added to culture. Fluorescence as Arbitrary Units (AU) was measured. (n = 6 wells per group). **D)** Total MMP activity (Abcam ab112146) in cell lysate were normalized to total protein in THP-1 cells treated with 100ng/mL IGF-1 for 24 hours (n = 4−5 wells per group). **E)** In independent experiments, Active MMP8/Total MMP8 levels were normalized to total protein in THP-1 cells treated with 100ng/mL IGF-1 for 24 hours and measured via QuickZyme MMP8 Activity Assay in media (n = 3 wells per group). **A,B** used 1-way ANOVA with a Tukey post hoc test. All other statistical tests are Student 2-tailed *t* test. *p < 0.05 vs control, **p < 0.01 vs control.

To study the effect of MMP8 downregulation on MF function, we utilized human THP-1 macrophages. IGF-1 reduced MMP8 levels in the conditioned media of THP-1 macrophages by 34.0 ± 1.2% (IGF-1, 100 ng/ml) compared to control cells (Fig 1A). The cyclolignan picropodophyllin (PPP) has been shown to be a potent and specific inhibitor of IGF-1 receptor (IGF-1R) phosphorylation and downstream signaling [42,43]**.** THP-1 pretreatment with PPP completely blocked the IGF-1 effect (Fig 1B) showing that IGF-1R mediated MMP8 downregulation. To test the potential effect of IGF-1 on collagenase activity, we measured collagenase activity in IGF-1-treated THP-1 cells utilizing fluorescent DQ collagen. IGF-1 induced a dramatic (>3-fold) reduction of collagenase activity in cell lysates compared to control (Fig 1C). While IGF-1 moderately lowered (16.2% ± 0.2%) the general activity of MMP enzymes which was assessed using a proprietary MMP substrate (Fig 1D), there was a trend IGF-1-dependent decreased MMP8 collagenase activity by 43 ± 1.2% (p = 0.077) in cell conditioned medium (Fig 1E), consistent with downregulation of MMP8 by IGF-1.

### Macrophage-specific IGF-1 overexpression decreases plaque MMP8 and downregulates pro-inflammatory cytokines in atherosclerotic mice

**Fig 2:**
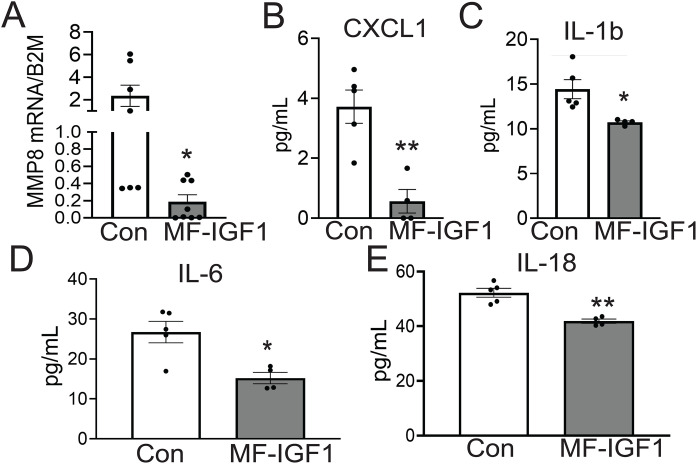
Macrophage-specific IGF-1 overexpression decreases plaque MMP8 and. **downregulates pro-inflammatory cytokines in peritoneal macrophages. A)** MMP8 mRNA levels in plaque in aortic root isolated by Laser Capture Microdissection (LCM) in MF-IGF1 and control (Apoe^-/-^) mice after 12 weeks on high fat diet (n = 7−8 mice per group). Beta-2 Microglobulin (B2M) serves as housekeeping gene. **B-E)** Peritoneal macrophages were isolated from MF-IGF1 and control mice. Cells were cultured overnight, and supernatant was collected. Legendplex beads bound to and quantified a number of inflammatory cytokines (n = 4 mice per group). All statistical tests are Student 2-tailed *t* test. *p < 0.05 vs control, **p < 0.01 vs control.

We have reported that mice with macrophage-specific IGF-1 overexpression (MF-IGF-1/Apoe^-/-^ mice) have decreased MMP8 levels in peritoneal MF compared to control Apoe^-/-^ mice [31,38]. In contrast, peritoneal MF from IGF-1R deficient mice have increased MMP8 levels [31,38]. For the current study we used laser capture microscopy (LCM) to isolate plaque tissue from the aortic root of MF-IGF-1/Apoe^-/-^ and control mice. We found a dramatic 92 ± 0.5% decrease in MMP8 mRNA levels in LCM isolates in MF-IGF-1/Apoe^-/-^ mice compared to control mice (Fig 2A). This result is consistent with MMP8 downregulation in peritoneal cells. Next, we isolated peritoneal macrophages from MF-IGF-1/Apoe^-/-^ mice and Apoe^-/-^ (control) mice and quantified expression of pro-inflammatory cytokines in conditioned medium. IGF-1-induced MMP8 downregulation was associated with decreased levels of CXCL1, IL-1b, IL-6, and IL18 cytokines (Fig 2B-E).

### MMP8 deficiency reduces MF inflammatory markers and promotes the M2 phenotype

**Fig 3:**
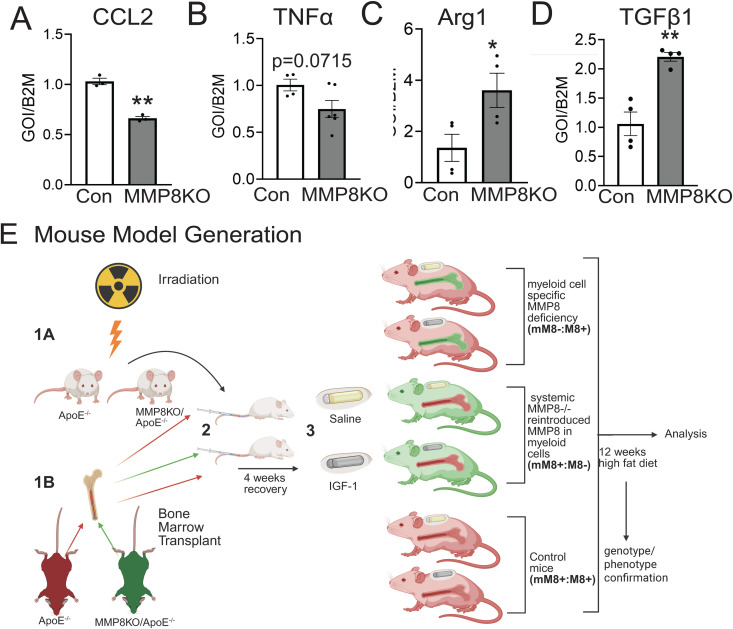
MMP8 deficiency polarizes macrophages toward an M2 phenotype. **A-D)** Peritoneal macrophages were isolated from MMP8-/- mice and Apoe^-/-^ (control) and cultured for 24 hours. RNA was extracted and mRNA levels were assessed for a number of polarization markers. (n = 4 mice per group). All statistical tests are Student 2-tailed *t* test. p < 0.05 vs control, **p < 0.01 vs control. **E)** Experimental design to generate mice with myeloid cell-specific MMP8 deficiency. Bone marrow transplantation and IGF-1 administration. Global MMP8 knockout mouse (Jackson Lab; B6.129X1-*Mmp8*^*tm1Otin*^/J 005514) were bred to Apoe^-/-^ background to promote atherosclerosis (MMP8KO/Apoe^-/-^ mice). 1A: At 8 weeks of age, MMP8KO/Apoe^-/-^ and Apoe^-/-^ mice were irradiated (9.5Gy) and 1B: bone marrow isolated from MMP8KO/Apoe^-/-^ and Apoe^-/-^ mice was introduced to irradiated mice via tail vein injection. 3: After four**-**week recovery, osmotic pumps (Alzet 2006) administrating 1.5 mg/kg/day of IGF-1 or solvent (control) were implanted for three months as the animals are fed a high fat western diet. Key: (mM8-:M8 + mice), atherosclerotic mice with cell specific MMP8 reintroduction (mM8+ :M8-) or control wild type MMP8 (mM8+ :M8 + , control).

The polarization of MF alters their functional phenotype during atherogenesis, where inflammation drives the process [44]. To test whether MMP8 deficiency alters MF polarization, we isolated peritoneal MF from MMP8-KO/Apoe^-/-^ mice and control mice and quantified expression levels of M1 (CCL2, TNFα) and M2 (ARG1, and TGFβ1) markers. MFs deficient in MMP8 have decreased levels of CCL2 (M1 marker) (33% ± 0.7% decrease vs. control, p < 0.01) a trend of decrease of TNFα (25.4% ± 0.6% vs control, p = 0.0715) and increased ARG1 (2.4-fold increase vs. control, p < 0.05), and TGFβ1 (3.2-fold increase vs. control, p < 0.005) (M2 markers) (Fig 3A-D), showing that MMP8 downregulation drives MF toward the M2 phenotype.

### Generation of myeloid cell specific MMP8 deficient mice

To study the effects of MMP8 deficiency in MF on atherosclerosis, we irradiated Apoe^-/-^ or MMP8KO/Apoe^-/-^ mice and performed bone marrow transplantation (BMT). Although this would affect all immune cells, as MF are the major myeloid cell present in plaque and MFs secrete MMP8 [45] (Fig 1), we hypothesized that BMT would result in MMP8 deficiency in MF in atherosclerotic plaque. We generated three groups of mice (Fig 3E):

1) Control mice: (mM8 + :M8+): Apoe^-/-^ mouse receiving WT MMP8 transplant (i.e., (Apoe^-/-^ bone marrow into Apoe^-/-^ mouse);
2) Myeloid MMP8-deficient: mice with MF-MMP8 deficiency (mM8-:M8+): Apoe^-/-^ mouse receiving BMT from MMP8KO/Apoe^-/-^ mice (i.e., MMP8KO/Apoe^-/-^ bone marrow into Apoe^-/-^ mouse);
3) Myeloid MMP8-rescued: mice with systemic MMP8 deficiency with reintroduced MMP8 in MF (mM8 + :M8-) (Apoe^-/-^ bone marrow into MMP8KO/Apoe^-/-^ mouse).

We confirmed successful BMT by genotyping DNA from tail and from whole blood. We found the expected genotype in all three groups (S1A Fig). We further confirmed that *Mmp8* gene deletion in bone marrow cells reduces MMP8 in the circulation by ELISA (S1B Fig). After recovery from BMT, we administered IGF-1 or saline (control) to each group of mice resulting in a total of 6 groups. All animals were fed with a high fat diet (HFD) for 12 weeks to accelerate atherosclerosis (Fig 3E). We confirmed that circulating IGF-1 was increased in IGF-1-treated mice by 44% ± 16% compared to saline-treated controls (S1C Fig). We found no change in body weight (BW) in IGF-1 treated mice compared to saline-treated mice (S2 Fig).

### MF- specific MMP8 deficiency reduced MMP8 and MF content in atherosclerotic plaque

**Fig 4:**
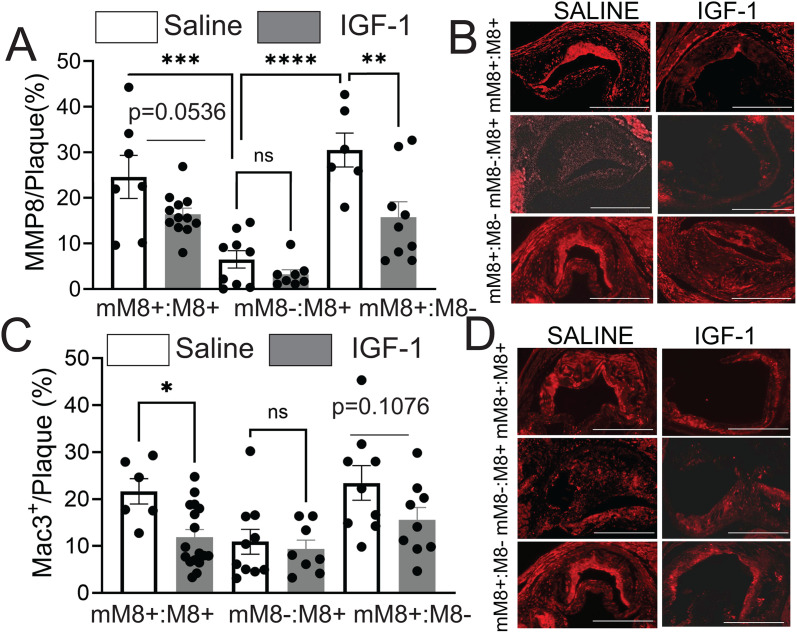
Myeloid cell specific MMP8 deficiency reduces plaque MMP8 and macrophage content. Serial aortic root cross-sections were stained with Mac3 or MMP8 antibody. **A,B)** Representative images and quantification of MMP8 in atherosclerotic plaque after IGF-1 treatment and high fat diet for 3 months. (n = 7–12 animals per group, scale bar 100µM). **C,D)** Representative images and quantification of MAC3 staining in atherosclerotic plaque after IGF-1 treatment and high fat diet for 3 months. N = 6–17 animals per group. Quantification is of area of positive staining normalized to area of plaque. Representative images are of all females. *p < 0.05, **p < 0.01, ***p < 0.005, ****p < 0.001. All statistics are One-way ANOVA with Tukey’s post-hoc test. Key: (mM8-:M8 + mice), atherosclerotic mice with cell specific MMP8 reintroduction (mM8 + :M8-) or control wild type MMP8 (mM8 + :M8 + , control).

It has been reported that systemic MMP8 deficiency markedly suppressed atherosclerosis development [18], however the effect of MF specific MMP8 on atherosclerosis has not been investigated. We found that mice with Myeloid MMP8-deficient (mM8-:M8 + mice) have significant (approximate 5-fold, p < 0.01) reduction of MMP8 levels in the plaque compared to controls (Fig 4A, B). These data suggest that MFs are a major source of plaque MMP8. IGF-1 produced a trend of reduced MMP8 levels in the atherosclerotic plaque cells in control mM8 + :M8 + mice (p = 0.0536) and in MMP8-deficient mice with Myeloid MMP8-rescued (mM8 + :M8-) (p < 0.005 vs. saline-treated control, Fig 4A,B). IGF-1-induced MMP8 downregulation in plaque was completely abolished by MF MMP8 deficiency (p = NS, IGF-1 vs. saline in Myeloid MMP8-deficient mice) showing that MF is the major source of MMP8 in a plaque which is negatively regulated by IGF-1. To evaluate a potential sex-related effect of IGF-1 on MMP8 levels, we analyzed data separately for males and females. IGF-1 reduced plaque MMP8 levels in female Myeloid MMP8-rescued (mM8 + :M8-) and control mice (both are P < 0.05) and MF MMP8 deficiency abolished this effect. IGF-1 did not significantly alter MMP8 levels in any male groups (S3B,C Fig).

Similarly, we found that IGF-1 treatment decreased macrophage presence in control mice (45.1% ± 6.6%, p < 0.005) similar to the levels in Myeloid MMP8-deficient (mM8-:M8+) mice (Fig 4C, D). We noted a trend of decrease in IGF-1 treated mice with reintroduced MMP8 (p = 0.1076) which becomes significant only in females (S4A-C Fig), suggesting sex-dependent effects.

### IGF-1 increases features of plaque stability only in mice with reintroduced MF MMP8 and does not affect atherosclerotic burden after irradiation

Systemic MMP8 deficiency decreased atherosclerosis in non-irradiated MMP8KO/ Apoe^-/-^ mice [18] and IGF-1 administration reduced atherosclerotic burden in Apoe^-/-^ mice [41]. For the current study we performed experiments with irradiated BMT mice to test the effect of MF MMP8 deficiency with/without IGF-1 on atherosclerosis and plaque morphology. Atherosclerotic burden was quantified in the entire aorta by *en face* analysis with Oil Red O staining and also by measuring lesions’ cross-sectional area in the aortic root. We found no difference in atherosclerotic burden in mice with macrophage-specific MMP8 deficiency (saline, Myeloid MMP8-deficient mM8-:M8+) compared to control mice (saline, control mM8 + :M8+) (S7A Fig, S8A Fig). To our surprise, IGF-1 caused virtually no change in atherosclerotic burden in control mice and only non-significant decrease in atherosclerosis in mice with MMP8 deficiency and with reintroduced MMP8 in myeloid cells (*En face* analysis, S7 Fig). We did not see any sex differences in burden (S7B,C Fig, S8B,C,D Fig).

**Fig 5:**
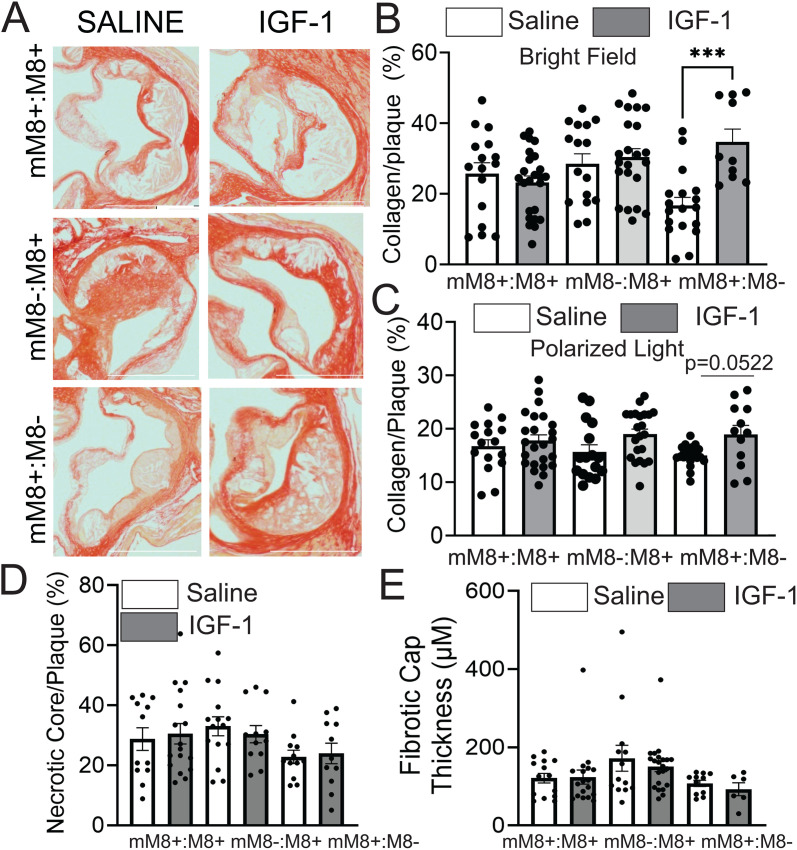
IGF-1 increases collagen in mice with myeloid MMP8 reintroduction. **A)** Aortic root sections were stained with Picrosirius Red and imaged under normal or polarized light. (n = 14–24 mice per group). **B,C)** Quantification of total Picrosirius staining normalized to plaque area IGF-1 treatment and high fat diet for 3 months. **D,E)** Quantification of necrotic core and average fibrotic cap. *p < 0.05, ***p < 0.005. All statistics are One-way ANOVA with Tukey’s post-hoc test. Representative images and quantification are of combined sexes. Key: (mM8-:M8 + mice), atherosclerotic mice with cell specific MMP8 reintroduction (mM8 + :M8-) or control wild type MMP8 (mM8 + :M8 + , control).

Plaque collagen level is a major determinant of plaque stability [46]. We visualized plaque collagen by staining with picrosirius red (PSR). PSR signal was quantified under bright field and polarized light microscopy. While PSR staining quantifies total collagen under bright field microscopy, examining PSR pattern under polarized light microscopy allows to distinguish mature/dense packed fibers (seen as red), intermediate fibers (yellow) and loosely packed collagen fibers (green) [47]. Under bright field microscopy, IGF-1 increased collagen only in mice lacking systemic MMP8 and Myeloid MMP8-rescued (p < 0.005, mM8 + :M8-; IGF-1, 35% ± 3% vs. saline 16% ± 3% collagen/plaque, sex combined data) (Fig 5A,B). Also, by quantification made under polarized light, the same group had a strong trend of collagen increase (p = 0.052) in IGF-1 treated animals (Fig 5C). Although we did not see any change in necrotic core size, we did see a slight trend increase of fibrotic cap thickness in mice with MF MMP8 deficiency (Fig 5D,E). The IGF-1 effect was lost in irradiated animals in other groups. Similar to our plaque MMP8 and macrophage data, we did note a sex difference, but only in animals lacking systemic MMP8 and Myeloid MMP8-rescued (mM8 + :M8-). IGF-1 induced almost 2-fold increase in total plaque collagen in Myeloid MMP8-rescued mice and this effect was significant (p < 0.0001) in females but not in males (S5,S6 Figs).

We have previously shown that IGF-1 increases plaque collagen and mature collagen fibers in non-irradiated Apoe^-/-^ mice [22]. IGF-1-induced collagen upregulation was also detected using PSR polarization microscopy (intermediate fibers) in female Myeloid MMP8-rescued mice (IGF-1, 25.49 ± 1.6% vs. saline 16.88 ± 0.7%) (S5C,D,E Fig) whereas there was no change in males (S6C,D,E Fig). Overall, our findings suggest that MF are the target of IGF-1 action, increasing collagen deposition within a plaque, mediated by downregulation of MMP8. Intriguingly, such the effect of IGF-1 was evident in female mice but not in male mice, suggesting a sex-dependent mechanism.

Thus, these results show that MF MMP8 deficiency does not change atherosclerotic burden in irradiated BMT mice, however plaques in mice with MF MMP8 deficiency have reduced MF and slightly increased collagen suggesting increased plaque stability, at least in females.

## Discussion

Here we show that IGF-1 reduces macrophage expression of MMP8 and MMP8 activity *in vitro*. We also found that MF IGF-1 overexpression reduces MMP8 levels in atherosclerotic plaque in a mouse model of atherosclerosis. In peritoneal macrophages from MMP8 deficient mice, we found a decrease of CCL2 expression and an increase in a number of anti-inflammatory markers. In parallel, peritoneal macrophages from MF-IGF1 mice express reduced inflammatory markers; together, it has been suggested that the down regulation of MMP8 may be at least a part of mechanisms whereby IGF-1 suppresses pro-inflammatory phenotype of MFs. We also found that MF specific MMP8 deficiency decreases macrophage content in atherosclerotic plaques. IGF-1 treatment in addition to MF-MMP8 deficiency did not cause further decrease of macrophage content, suggesting that the ability of IGF-1 to decrease plaque macrophage content is mediated through downregulation of MMP8. Interestingly, irradiation appeared to block the ability of IGF-1 to reduce atherosclerotic burden and to increase plaque collagen, although the collagen effect was maintained in female mice with myeloid specific MMP8 expression on a background of systemic MMP8 deficiency. Because no validated mouse model of MMP8 flox mouse exists, we decided to use bone marrow transplant as a model of MF-MMP8 deficiency. Although irradiation limits translation to non-irradiated models, cancer patients (pan-cancer) die of atherosclerosis at a higher rate than non-cancer patients, and this is accelerated in patients that have aggressive cancers [48].

MMP8, first identified in neutrophils, is shown to be increased in macrophages in atherosclerosis [23,31]. Of note, MMP8 has been found to be increased in vulnerable plaques in both humans and mice [18]. There are other MMPs implicated in atherosclerosis, but MMP8 is one of the only MMPs that has similar levels of expression in macrophages in both humans and mice [49]. We have previously shown that macrophages overexpressing IGF-1 have lower expression levels of MMPs, including MMP8 [31]. Here we recapitulate that effect in human cells. Because MMP8 exists in pro and active forms, we also wanted to understand if a reduction in expression would result in a reduction in activity. Indeed, we show a reduction in both total collagenase activity and MMP8 activity in human THP-1 macrophages treated with IGF-1.

Recent studies have shown that while residential macrophages in healthy (mouse) aorta are fairly homogenous, multiple transcriptomes of plaque macrophages have been described [50], and a recent study identified six unique clusters in human atheroma [51]. *In vitro*, it has been shown that some macrophage phenotype switching is possible, and this likely occurs *in vivo* [11]. Because we demonstrated a reduction of MMP8 in plaque by macrophage specific IGF-1 overexpression, we were prompted to investigate if MMP8 changes macrophage behavior. Classically, inflammatory and pro-atherogenic macrophages are designated as M1 and reparative/anti-inflammatory are designated as M2 [12,13]. This distinction is now considered incomplete in most disease models, and in the case of atherosclerosis, the polarization of MF alters their functional phenotype in response to atherogenesis [44], however the distinction remain useful as the heterogenous population of plaque macrophages are grouped in a spectrum from M1 to M2 [52]. It has been shown also that the stability of atherosclerotic plaques depends on the quantity and quality (i.e., polarization state) of infiltrated MF [44]. Alternatively activated M2 MF, polarized locally or recruited from the circulation, produces anti-inflammatory cytokines such as IL-10 and Transforming Growth Factor β (TGFβ1) which stimulates collagen expression [5]. M2 MF have been shown to have roles in resolving injury and in angiogenesis [52]. Of note, murine models develop early and intermediate atherosclerotic lesions, but this does not lead to plaque rupture seen in human disease. It is possible that different stages have different populations of macrophages, which requires more study [53,54]. Although macrophage polarization is beyond the scope of this study, there has been only one study showing MMP8 effects on macrophage polarization, suggesting that MMP8 polarizes MF towards a more M2 phenotype [20]. In that study, the authors found that MMP8 induces M2 polarization by degrading fibromodulin, increasing the bioavailability of TGFβ1 [20]. These data were shown *in vitro*, which de facto does not take into account potential modulatory signaling effects from other plaque components. Here we report the opposite effect, which is taken in the context of the heterogeneity of atherosclerotic plaque, highlights the complexity of macrophage regulation. Further, MMP8 has been shown to promote atherosclerosis by activating Angiotensin II, increasing inflammation [18], highlighting other *in vivo* targets to be assessed in future studies.

A previous study showed that global knockout of MMP8 results in increased collagen deposition and reduced atherosclerosis [18]. MMP8 is expressed by many plaque cell types [23], but as macrophages are the major source of MMP in atherosclerosis, we first wanted to investigate the role of MMP8 derived from macrophages and how IGF-1 influences MF phenotype. For this purpose, we irradiated mice and utilized bone marrow transplant to understand how myeloid derived MMP8 and IGF-1 affects atherosclerosis. We demonstrated that the deficiency of MMP8 in macrophages leads to a reduction in both the expression level of MMP8 and the presence of macrophages in atherosclerotic lesions.

MF MMP8 deficiency dramatically decreased plaque macrophages compared to control mice and macrophage content was restored by reintroduction of MMP8 in MFs (Fig 4C,D), suggesting that MF MMP8 is involved in recruitment and retention of plaque MFs. Control mice and mice with reintroduced MF MMP8 treated with IGF-1 had lower plaque MF content (Control, p < 0.05, reintroduced MMP8, p = 0.1076) (Fig 4 C,D). IGF-1-induced decrease in plaque MF was completely blocked in mice with MF MMP8 (mMF8-:M8+) deficiency indicating that IGF-1 mediated downregulation of plaque MFs is MMP8 dependent.

Collagen plays a key role in determining plaque stability [46]. We visualized plaque collagen by staining it with picrosirius red (PSR). In our previous findings, we found IGF-1 increased plaque collagen and matured fibers in non-irradiated Apoe^-/-^ mice [22]. In the current experiment, we found only a trend of small increase in total collagen in irradiated IGF-1-treated control females. However, IGF-1 induced almost a 2-fold increase in total plaque collagen in Myeloid MMP8-rescued mice (sex combined data) in both brightfield and PSR imaging (Fig 5B,C) and this effect was significant (p < 0.001) in females but not in males (S5,S6 Figs). IGF-1-induced collagen upregulation was also detected using PSR polarization microscopy (intermediate fibers) in female Myeloid MMP8-rescued (mM8 + :M8-) mice, suggesting that myeloid cells play essential roles in mediating the IGF-1 effect on plaque collagen. Of note, the apparent sex-specific effect of IGF-1 post irradiation is hypothesis generating and will need to be explored in future studies looking at potential sex differences in the ability of IGF-1 to regulate collagen levels. Collagen levels were not changed in the absence of myeloid MMP8 or presence of global MMP8 (Fig 5), suggesting possible compensation by other collagenases.

Interestingly, atherosclerotic burden was unchanged in any group, likely due to the impact of irradiation on atherosclerotic progression. It has been noted that irradiation accelerates atherosclerosis in both mice and humans [55]. Radiation treatment causes cardiovascular disease in patients in the form of accelerated atherosclerosis [56], especially in accelerating coronary artery disease [57]. Schiller et al. established that irradiated BMT mice have a distinct phenotype of atheroma compared to non-irradiated controls featuring increased lesion size and reduced plaque collagen [55]. It was also reported that BMT mice exhibit impaired endothelial responses in regions (such as aortic sinus) that are more prone to the detrimental effects of disturbed flow and via this mechanism, radiation may promote atherogenesis. Of note, murine models develop early and intermediate atherosclerotic lesions, but this does not lead to plaque rupture seen in human disease. It is possible that different stages have different populations of macrophages, which requires more study [53,54]. We and others have demonstrated IGF-1-induced reduction in atherosclerotic burden in non-irradiated Apoe^-/-^ [39,41,58]. We speculate that irradiation/BMT procedure-induced accelerated atherogenesis prevented the IGF-1-induced anti-atherogenic effect. Our finding that IGF-1 effects on plaque MMP8, MF content and collagen occurred only in mice with systemic MMP8 deficiency and myeloid MMP8 reintroduction suggests that MF MMP8 is the main mediator of IGF-1 increasing collagen content in atherosclerotic plaque. However, it is possible that other MMP8 expressing cells may mask the effect of macrophage-specific MMP8 downregulation during atherosclerotic plaque development and plaque stability in the context of irradiation. These mechanisms and interpretations need to be further examined. Interestingly, there are conflicting reports in rodents if females show more protective features against atherosclerosis after irradiation compared to males [28,59]. In humans, females have more cardiovascular events than males after radiation therapy, although they have improved outcomes [60]. Further, there has been limited studies that implicate that estrogen and other sex hormones have a role in regulating MMP8 and other MMP expression [61,62], which may explain the sex-specific effects in this study. Of note, rats that had ovariectomy showed increased MMP8 expression in bone [63]. Coupled with research showing that (ultraviolet) irradiation increases MMP8 expression in human derma, it may be that estrogen protects against irradiation mediated MMP8 elevation which masks IGF-1’s effect in males [64].

This study suggests that one of IGF-1’s atheroprotective mechanisms is to limit macrophage accumulation via downregulation of MMP8 expression in plaque. In fact, MF specific MMP8 deficiency dramatically decreased plaque macrophages compared to control mice and macrophage content were restored by reintroduction of MMP8 in myeloid cells (Fig 4C, D). IGF-1 downregulated plaque MF in control mice and in mice with reintroduced MMP8 in myeloid cells (Fig 4 A, B). IGF-1-induced decrease in MF was completely blocked in mice with MF cell specific MMP8 deficiency showing that MF cell MMP8 mediates the IGF-1 effect on plaque MF. These data indicate that MF MMP8 has a role in regulating MF content in plaque, mechanisms that need to be further investigated. Coupled with previous findings from our group [31,38,41], our data suggests that IGF-1 modulation of MF activity plays an important role in its atheroprotective features.

Our findings demonstrate that IGF-1 downregulates MMP8, which establishes a potentially important mechanism whereby macrophage-derived IGF-1 may have significant anti-atherosclerotic effects, suggesting that MMP8 may provide a potential new therapeutic target. However, given MMP8’s complex role in inflammation and tissue remodeling, cell specific MMP8 regulation requires further study.

## Conclusion

We found that absence of MMP8 drives macrophage polarization towards a less inflammatory phenotype. Our results show that myeloid cell specific MMP8 deficiency does not change atherosclerotic burden in irradiated BMT mice, however it reduced MF and increased collagen content in plaques only in female mice, consistent with increased plaque stability. IGF-1 can negatively regulate MMP8 expression in MF, and MMP8 deficiency omitted the IGF-1’s effects of increasing collagen contents. However, irradiation introduced unexpected confounding effects. Further investigation, involving the creation of an MF MMP8 knockout mouse model (Cre-lox system or CRISPR) can clarify our interpretation here. Nevertheless, these results support a mechanism of IGF-1 enhancing plaque stability by downregulating MMP8 in MF. Overall, our results suggest the importance of macrophage MMP8 as a potential target to treat unstable atherosclerosis.

## Materials and methods

### Materials

Recombinant human IGF-1 (Increlex) was from IPSEN. Immunoblotting, immunohistochemistry and quantitative real-time PCR analysis were performed as described previously [38]. Antibodies used include Anti-CD107b (Mac3, Biolegend 105502) and MMP8 (Novus AF3245). Quantification is of area of positive staining normalized to area of plaque. ELISAs were used to measure human IGF-1 (RnD DG100B), mouse IGF-1 (RnD MG100), and MMP8 (pro-peptide and active, abcam ab206982). Legendplex beads (740845) were used as described by the manufacturer to quantify chemokines. Total MMP activity, MMP8 activity, and collagenase activity was quantified by MMP Activity Assay Kit (Abcam ab112146), MMP8 Activity Assay (QuickZyme, QZBMMP8H) and DQ Collagen (Thermo D12060) fluorescence, respectively.

### Animals

All animal experiments were approved by the Institutional Animal Care and Use Committees of Tulane University. Apoe^-/-^ mice (C57BL/6) were received from Jackson Lab. MMP8 knockout mouse were cryorecovered by Jackson Lab (B6.129X1-*Mmp8*^*tm1Otin*^/J 005514) and bred to an Apoe^-/-^ background. Animals were genotyped using the following primers: 5’ CTT TCA ACC AGG CCA AGG TA3’; 5’ CAC GAG ACT AGT GAG ACG TG3’; 5’GCC CTT AAA CCG CTA AGG AG3’. Apoe^-/-^ mice were bred in parallel as controls. MF-IGF1/Apoe^-/-^ mice were generated as we previously described [31]. Apoe^-/-^ littermates were used as controls. Mice were housed individually and maintained on a 12-hour light–dark cycle. Mice were fed with high-fat diet (HFD), infused with saline or IGF-1 (1.5 mg/kg/d) as we previously described [40]. Data was analyzed in combination or separated by sex.

### Cultured cells

THP-1 monocytes were differentiated into macrophages using phorbol myristate acetate (PMA, 25ng/mL) for 48 hours. Cells were plated at 1.2x10^6^ cells/well, 6 well plate. Cells were incubated in serum free media for 24 hours before any treatment. IGF-1 was administered to cells at increasing concentrations (20 and 100ng/mL).

### Bone marrow transplant

Eight-week-old animals were irradiated with 9.5 Grays utilizing a GammaCell40. Bone marrow was isolated from donor animals euthanized by CO_2_ and cervical dislocation. Sexes were matched between donor and recipient. Bone marrow was isolated from femur and tibia and cells were injected into recipient mice via tail vein injection (500,000 cells in 200μl of media per mouse). Next, Apoe^-/-^ or MMP8KO/ Apoe^-/-^ were irradiated and reintroduced bone marrow, generating three groups: control mice (mM8 + :M8+): Apoe^-/-^ mouse receiving WT MMP8 transplant (i.e., Apoe^-/-^ bone marrow into Apoe^-/-^ mouse); mice with Myeloid MMP8-deficient (mM8-:M8+): Apoe^-/-^ mouse receiving BMT from MMP8KO/ Apoe^-/-^ mice (i.e., MMP8KO/Apoe^-/-^ bone marrow into Apoe^-/-^ mouse); mice with systemic MMP8 deficiency with reintroduced MMP8 in myeloid cells (Myeloid MMP8-rescued, mM8 + :M8-) (Apoe^-/-^ bone marrow into MMP8KO/ Apoe^-/-^ mouse). After four-week recovery in sterile conditions, animals were placed on a high fat western diet (Envigo TD.88137) for 12 weeks. At the time of diet, osmotic pumps (1.5 mg/kg/day Alzet 2006) were surgically implanted. Pumps had a duration of 6 weeks, and as such, pumps were reimplanted at the 6-week point. Body weight (BW) was monitored every two weeks and any animal dropping >20% BW was euthanized (S2 Fig). Bone marrow transplant was confirmed by detecting MMP8 presence or deletion in circulating leukocyte genomic DNA. Leukocyte DNA extraction was performed using New England Biolabs Monarch Spin gDNA Extraction kit according to manufacturer’s instructions.

### Laser capture microdissection (LCM)

Aortic root sections were isolated and dissected as previously described [21,65].

### Isolation of peritoneal macrophages

Peritoneal macrophages were isolated as previously described [38]. Briefly, peritoneal macrophages were elicited from MMP8 KO and Apoe^-/-^ mice by injection of 10% thioglycolate broth, then peritoneal lavage elicited cells. Macrophages were enriched by adhesion to a 6 well plate and cultured overnight. These cells were assessed via western blot for MMP8 expression [31] and via RT-PCR (for polarization markers).

### Atherosclerosis quantification

After 12 weeks of high fat diet, animals were sacrificed, and atherosclerosis was quantified as previously described [41]. Briefly, 6μm serial sections through the aortic root and stained with hematoxylin and eosin. The mean value of plaque cross-sectional areas from 3 sections was used for quantification. *En face* images were acquired using a Leica EZ4 and aortic root images were acquired using an Olympus IX71 (DP80 camera). All images were analyzed using CellSens Dimension (Olympus) Plaque composition was assessed by immunostaining of aortic root cross-sections for Mac3 and MMP8. Picrosirius red staining was used to quantify collagen under bright field and polarized light. Necrotic core was analyzed by the absence of staining in Picrosirius red stained roots. Fibrotic cap thickness was measured from the end of the necrotic core to the end of positive Picrosirius staining along 10µM segments in each plaque. All IgG controls for immunohistochemistry data can be found in S9 Fig.

### Statistical analysis

All numeric data are expressed as mean±SEM. Statistical analyses were performed with GraphPad PRISM (version 8.0) software. Significant differences were determined by unpaired Student *t-* test with or without the Welch correction, or one-way ANOVA with either a Dunnett’s or Tukey’s post hoc test accordingly with the normality of residuals distribution or sample size. Fisher’s exact test was used to compare frequency distribution differences between groups. The exact test used is mentioned in every figure legend. Differences were considered significant at *P* < 0.05. We declare that the design, execution, and reporting of the current study adheres to the guidelines for experimental atherosclerosis studies described and recommended by the American Heart Association, and we also considered sex as a biological variable as explained by the ATVB Council [66,67].

## Supporting information

S1 Fig Generation of myeloid cell specific MMP8 deficient mice. **A)** Representative image showing macrophage specific deletion or reintroduction of MMP8. After irradiation and bone marrow transplant (4 weeks), whole blood and tail snips were collected from mice. DNA was isolated from whole blood which showed deletion of MMP8, while tail samples showed whole body presence or absence of MMP8. **B)** Protein levels of MMP8 in serum were measured in irradiated and transplanted mice at sacrifice. (n = 5−8 mice per group). Mice without MF MMP8 had significantly lowered levels of circulating MMP8 compared to all other groups. **C)** At sacrifice, IGF-1 levels (mouse, human (infused), and total) were measured in serum by ELISA (n = 11–12 mice per group from all BMT transfer groups). *p < 0.05, **p < 0.01, ***p < 0.005, ****p < 0.001. **B)** All statistics are One-way ANOVA with Tukey’s post-hoc test. **C)** All statistics are two tailed t-test. Control mice (mM8 + :M8+): Apoe^-/-^ mouse receiving WT MMP8 transplant. (mM8-:M8+): mice with MF-MMP8 deficiency in Apoe^-/-^ mouse. (mM8 + :M8-) mice with systemic MMP8 deficiency with reintroduced MMP8 in MF. Key: (mM8-:M8 + mice), atherosclerotic mice with cell specific MMP8 reintroduction (mM8 + :M8-) or control wild type MMP8 (mM8 + :M8 + , control).
(EPS)

S2 Fig Body weight of mice with myeloid cell specific MMP8 deficiency. Mice weights were measured every two weeks after recovery from irradiation and bone marrow transplant. (n = 17–22 mice per group). Key: (mM8-:M8 + mice), atherosclerotic mice with cell specific MMP8 reintroduction (mM8 + :M8-) or control wild type MMP8 (mM8 + :M8 + , control).
(EPS)

S3 Fig MMP8 deficiency and IGF-1 have differential effects in males and females in MMP8 plaque levels. **A)** Quantification of MMP8 normalized to plaque area in female mice after IGF-1 treatment and high fat diet for 3 months (n = 3–8 mice per group. **B,C)** Representative images and quantification of MMP8 in males after IGF-1 treatment and high fat diet for 3 months (n = 3–7 mice per group). *p < 0.05, **p < 0.01, ***p < 0.005, ****p < 0.001. All statistics are One-way ANOVA with Šidák’s multiple comparisons test. Scale bar = 200uM. Key: (mM8-:M8 + mice), atherosclerotic mice with cell specific MMP8 reintroduction (mM8 + :M8-) or control wild type MMP8 (mM8 + :M8 + , control).
(EPS)

S4 Fig MMP8 deficiency and IGF-1 have differential effects in males and females in macrophage plaque content. **A)** Quantification of Mac3 normalized to plaque area in female mice after IGF-1 treatment and high fat diet for 3 months (n = 2–12 mice per group). **B,C)** Representative images and quantification of Mac3 in males after IGF-1 treatment and high fat diet for 3 months (n = 3–9 mice per group). *p < 0.05. All statistics are One-way ANOVA with Šidák’s multiple comparisons test. Scale bar = 200 µm. Key: (mM8-:M8 + mice), atherosclerotic mice with cell specific MMP8 reintroduction (mM8 + :M8-) or control wild type MMP8 (mM8 + :M8 + , control).
(EPS)

S5 Fig IGF-1 increases mature collagen in females in mice with systemic MMP8 deficiency and myeloid MMP8 reintroduction. Cross-sections of aortic root atherosclerotic plaque in female mice after BMT and 3 months high fat diet. Cross-sections were stained with picrosirius red, which stains thick, tightly packed collagen fibrils *red/orange*, intermediate fibrils *yellow*, and thin, loosely packed fibrils *green*. **A)** Representative images of valve sections at 90º rotation under polarized light. **B)** Quantification of total collagen in plaque in female mice. **C)** Quantification of red/orange fibers. **D)** Quantification of yellow fibers. **E)** Quantification of green fibers. (n = 5−12 mice per group). Green fibers are significantly decreased (p = 0.0268) in IGF-1 treated mice that have MMP8 only in MF. **p < 0.01, ****p < 0.001. All statistics are One-way ANOVA with Tukey’s multiple comparisons test. Scale bar = 100uM. All representative images and quantification are in females. Key: (mM8-:M8 + mice), atherosclerotic mice with cell specific MMP8 reintroduction (mM8 + :M8-) or control wild type MMP8 (mM8 + :M8 + , control).
(EPS)

S6 Fig IGF-1 does not increase collagen in males. Cross-sections of aortic root atherosclerotic plaque in male mice after BMT and 3 months high fat diet. Cross-sections were stained with picrosirius red, which stains thick, tightly packed collagen fibrils *red/orange*, intermediate fibrils *yellow*, and thin, loosely packed fibrils *green*. **A)** Representative images of root sections at 90º rotation under polarized light. B) Quantification of total collagen in plaque in male mice. **C)** Quantification of red/orange fibers. D) Quantification of yellow fibers**. D)** Quantification of green fibers. (n = 4–12 mice per group). All statistics are One-way ANOVA with Tukey’s multiple comparisons test. Scale bar = 100uM. All representative images and quantification are in males (females are in S5 Fig). Key: (mM8-:M8 + mice), atherosclerotic mice with cell specific MMP8 reintroduction (mM8 + :M8-) or control wild type MMP8 (mM8 + :M8 + , control).
(EPS)

S7 Fig IGF-1 has no effect on plaque burden on irradiated mice. Upon sacrifice, whole aortas were excised and stained with Oil Red O for *enface* atherosclerosis assessment. **A)** Quantification of atherosclerotic burden (plaque area/total aortic area in all mice (n = 11–16 mice). **B, C)** Quantification and representative images of female and males. **D)** Representative images of male mice. Key: (mM8-:M8 + mice), atherosclerotic mice with cell specific MMP8 reintroduction (mM8 + :M8-) or control wild type MMP8 (mM8 + :M8 + , control).
(EPS)

S8 Fig IGF-1 has no effect on plaque burden on irradiated mice. **A)** Quantification of aortic roots stained with H&E-stained assess lesional area normalized to total root area (n = 7–11 mice per group). **B**, **D)** Quantification and representative images of females (n = 4–6 mice per group). **C,E)** Quantification and representative images of males (n = 3–7 mice per group). Scale bar, 200 µm. Key: (mM8-:M8 + mice), atherosclerotic mice with cell specific MMP8 reintroduction (mM8 + :M8-) or control wild type MMP8 (mM8 + :M8 + , control).
(EPS)

S9 Fig Representative IHC IgG controls. A. IgG for rabbit MMP8 antibody. Scale bar, 200 µm. Mice were 16–20 weeks old on normal chow.
(EPS)

S1 File Raw data. (XLSX)

S2 File Supporting Information unmodified gel image Suppl Fig 1. (TIF)
