# Supplementary figures and images for: IGF-1 downregulates matrix metalloproteinase 8 to promote plaque stability: Evidence from myeloid cell-specific MMP8 in atherosclerosis
Source: PLoS One. 2025 Sep 24;20(9):e0332660. doi: 10.1371/journal.pone.0332660 (PMC12459808; doi:10.1371/journal.pone.0332660)

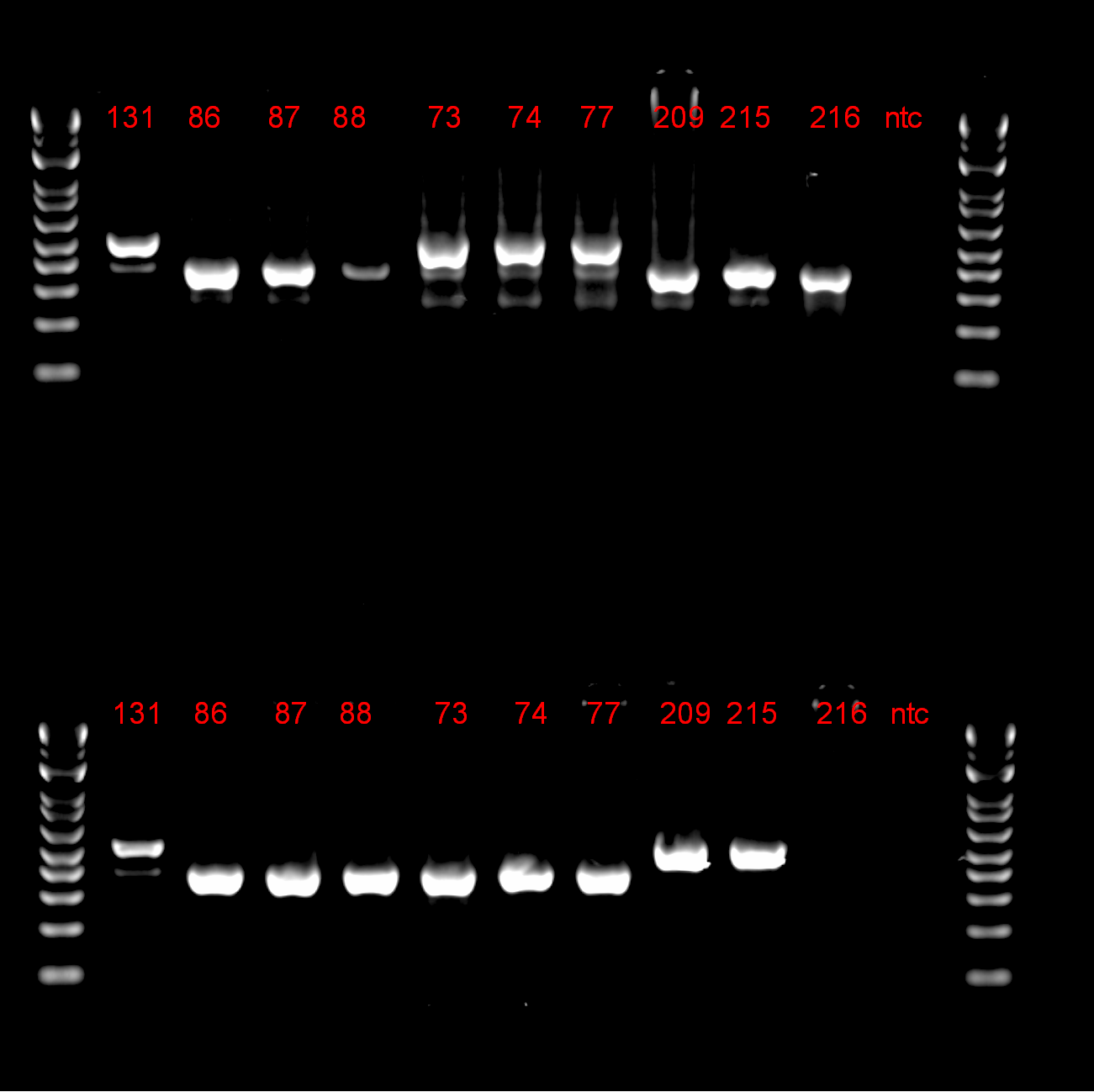

Supplement: S2 File — (TIF) [file pone.0332660.s011.tif]
